# The diabetes gene *Zfp69* modulates hepatic insulin sensitivity in mice

**DOI:** 10.1007/s00125-015-3703-8

**Published:** 2015-08-01

**Authors:** Bomee Chung, Mandy Stadion, Nadja Schulz, Deepak Jain, Stephan Scherneck, Hans-Georg Joost, Annette Schürmann

**Affiliations:** Department of Experimental Diabetology, German Institute of Human Nutrition Potsdam-Rebruecke, Arthur-Scheunert-Allee 114-116, D-14558 Nuthetal, Germany; German Center for Diabetes Research (DZD), Neuherberg, Germany; Institute of Metabolic Physiology, Heinrich Heine University of Düsseldorf, Universitätsstrasse, 1, D-40225 Duesseldorf, Germany

**Keywords:** Diabetes, Hepatosteatosis, Insulin resistance, Lipid metabolism, *Zfp69*

## Abstract

**Aims/hypothesis:**

*Zfp69* was previously identified by positional cloning as a candidate gene for obesity-associated diabetes. C57BL/6J and New Zealand obese (NZO) mice carry a loss-of-function mutation due to the integration of a retrotransposon. On the NZO background, the *Zfp69* locus caused severe hyperglycaemia and loss of beta cells. To provide direct evidence for a causal role of *Zfp69*, we investigated the effects of its overexpression on both a lean [B6-Tg(*Zfp69*)] and an obese [NZO/B6-Tg(*Zfp69*)] background.

**Methods:**

*Zfp69* transgenic mice were generated by integrating the cDNA into the ROSA locus of the C57BL/6 genome and characterised.

**Results:**

B6-Tg(*Zfp69*) mice were normoglycaemic, developed hyperinsulinaemia, and exhibited increased expression of *G6pc* and *Pck1* and slightly reduced phospho-Akt levels in the liver. During OGTTs, glucose clearance was normal but insulin levels were significantly higher in the B6-Tg(*Zfp69*) than in control mice. The liver fat content and plasma triacylglycerol levels were significantly increased in B6-Tg(*Zfp69*) and NZO/B6-Tg(*Zfp69*) mice on a high-fat diet compared with controls. Liver transcriptome analysis of B6-Tg(*Zfp69*) mice revealed a downregulation of genes involved in glucose and lipid metabolism. Specifically, expression of *Nampt, Lpin2, Map2k6, Gys2, Bnip3, Fitm2, Slc2a2*, *Ppargc1α* and *Insr* was significantly decreased in the liver of B6-Tg(*Zfp69*) mice compared with wild-type animals. However, overexpression of *Zfp69* did not induce overt diabetes with hyperglycaemia and beta cell loss.

**Conclusions/interpretation:**

*Zfp69* mediates hyperlipidaemia, liver fat accumulation and mild insulin resistance. However, it does not induce type 2 diabetes, suggesting that the diabetogenic effect of the *Zfp69* locus requires synergy with other as yet unidentified genes.

**Electronic supplementary material:**

The online version of this article (doi:10.1007/s00125-015-3703-8) contains peer-reviewed but unedited supplementary material, which is available to authorised users.

## Introduction

Multiple studies have demonstrated the shared contribution of both genetic and environmental factors in the development of type 2 diabetes mellitus [1, 2]. Genome-wide association and linkage studies have markedly improved our understanding of the genetic basis of type 2 diabetes in humans [3–5] and rodents [6–10], respectively. In mouse studies, diabetogenic quantitative trait loci (QTL) have been mapped by intercross of inbred strains with diabetes-related phenotypes, leading to the identification of several candidate genes for diabetes susceptibility or resistance [6, 8–10].

In an intercross of NON (non-obese non-diabetic) and NZO (New Zealand obese) mice, Leiter et al mapped the NON-derived diabetogenic locus *Nidd1* to chromosome 4 [8]. This locus contributed substantially to the development of hyperglycaemia and hypoinsulinemia [8]. A diabetogenic locus partially overlapping with *Nidd1* (*Nidd/SJL*) was identified in a backcross population of Swiss Jim Lambert (SJL) and NZO mice. The SJL-derived QTL caused severe hyperglycaemia and hypoinsulinaemia [9]. Moreover, when combined with the obesity QTL, *Nob1*, the diabetogenic effect from *Nidd/SJL* was greatly enhanced by a high-fat diet (HFD), which strongly suggests that *Nidd/SJL* contains a gene for obesity-associated diabetes [9].

By sequencing and gene expression profiling of the critical region of *Nidd/SJL*, we identified the zinc finger domain transcription factor *Zfp69* as the most likely candidate gene within the QTL. Mouse strains such as SJL and NON carry the diabetogenic allele of *Zfp69*, which generates a normal full length mRNA comprising a Krüppel-associated box (KRAB) and a Znf-C2H2 domain [11]. By contrast, carriers of the retrotransposon IAPLTR1a in intron 3 of *Zfp69* (NZO, C57BL/6J) produce a truncated mRNA and are less diabetes prone (NZO) or fully protected (C57BL6/J) [11].

In order to provide additional evidence for a causal role of *Zfp69* and to investigate the mechanism of its diabetogenic potency, we generated a transgenic mouse line overexpressing the gene on the B6 and NZO × B6 background, and studied glucose homeostasis and fat distribution. *Zfp69* induced the accumulation of liver fat and a mild insulin resistance, confirming the role of *Zfp69* as a diabetogenic gene.

## Methods

### Generation of a transgenic mouse line overexpressing *Zfp69*

*Zfp69* cDNA tagged with a C-terminal Myc epitope was fused to the ubiquitin C promoter. For integration into the ROSA locus, the construct was flanked by fragments corresponding with the sequence of this locus. A *Zfp69* transgenic mouse line was generated with C57BL/6J mice as background strain (Ozgene, Perth, Western Australia, Australia). To generate obese NZO/B6 F1 hybrid mice, B6-Tg(*Zfp69*) hemizygote male mice were mated with NZO/HIBomDife female mice (R. Kluge, German Institute of Human Nutrition, Nuthetal, Germany).

The animals were housed in a controlled environment (20 ± 2°C, 12 h/12 h light/dark cycle), fed a standard diet (SD; V153x R/M-H, Ssniff, Soest, Germany) or a HFD (45% energy from fat, D12451, Research Diets, New Brunswick, NJ, USA). All animal experiments were approved by the ethics committee of the State Office of Environment, Health and Consumer Protection (State of Brandenburg, Germany).

### Study design

Male mice [B6-wild-type (WT) and B6-Tg(*Zfp69)*; NZO/B6-WT and NZO/B6-Tg(*Zfp69)*] were fed SD or HFD. Body weight and blood glucose levels were measured weekly from 4 to 16 weeks of age, and then every other week until 24 weeks of age. Body composition was analysed at 8 and 16 weeks of age by computed tomography (CT). OGTT was performed at week 18. Animals were killed in a postprandial state at 24 weeks of age and plasma insulin and pancreatic insulin content were estimated.

### Quantitative real-time PCR

Total RNA was extracted and cDNA synthesis was performed as described previously [12] for quantitative real-time PCR (qPCR) via the LightCycler 480 system and FastStart Universal probe Master mix (Roche, Mannheim, Germany). Primers are listed in Electronic Supplementary Material (ESM) Table 1.

### Nuclear extract preparation and western blotting

Liver samples (8 weeks) were homogenised and nuclear extracts were isolated by a kit according to manufacturer’s instructions (Thermo Scientific NE-PER, Bonn, Germany). Nuclear extracts were analysed by western blot with a primary antibody against ZFP69 [11]. For the detection of phospho-Akt (pAKT) and total-Akt (tAKT), liver lysates were analysed by western blot with antibodies against pAKT, tAKT, both raised in rabbit, at 1:1,000 dilution (Merck Millipore, Darmstadt, Germany) and β-actin raised in mouse at 1:5,000 dilution (Sigma, Munich, Germany).

### Plasma analysis

Blood glucose was measured with a Glucometer Elite (Bayer, Leverkusen, Germany). Insulin was measured with ELISAs from DRG Diagnostics (Marburg, Germany), and triacylglycerols were measured with Triglyceride Reagent from Sigma. Measurements were performed in a blinded manner.

### Pancreatic insulin content and isolation of islets of Langerhans

Detection of total pancreatic insulin, isolation of pancreatic islets and detection of glucose-stimulated insulin secretion were determined as previously described [13].

### Quantification of beta cell area

Pancreases were fixed in 4% paraformaldehyde for 24 h. Evenly spaced 12 μm sections were stained for insulin (DAKO, Hamburg, Germany) to determine the beta cell area. Secondary Cy3-conjugated antibodies (Life Technologies, Darmstadt, Germany) and DAPI (Sigma) for cell-nuclei were used. The cross-sectional and insulin-positive areas were quantified using Fiji/ImageJ (Fiji, Dresden, Germany). Relative insulin-positive area was determined by quantification of cross-sectional insulin-positive area divided by cross-sectional area of the whole pancreas and presented as 100% of weight.

### OGTT

B6 mice were fasted for 6 h prior to an oral or intraperitoneal application of glucose (20% solution, 2 g/kg body weight), while NZO/B6 were fasted for 16 h to reach basal glucose levels before the application. Glucose and insulin concentrations were detected at indicated time points.

### Insulin tolerance test

For the insulin tolerance test (ITT), B6-WT and B6-Tg(*Zfp69*) mice (6 h fasted) were intraperitoneally injected with insulin (1 U for SD; 1.25 U for HFD) and the blood glucose levels were estimated from the tail-tips.

### Immunohistochemistry

Paraffin sections of the liver of B6-WT and B6-Tg(*Zfp69*) mice were prepared as described earlier [14]. Sections were incubated with anti-Plin2 antibody (Progen Biotechnik, Heidelberg, Germany) in combination with fluorescence-conjugated Alexa488-antibody (Life Technologies) and analysed with a Leica TCS SP2 Laser Scan inverted microscope (Leica, Wetzlar, Germany).

### CT

CT was performed using LaTheta LCT-200 (Hitachi-Aloka, Tokyo, Japan). Subcutaneous fat and visceral fat of B6-WT and B6-Tg(*Zfp69*) at 8 and 16 weeks of age on SD were determined as previously described [15].

### Liver fat

Liver samples were ground in liquid nitrogen and dissolved in HB buffer (10 mM NaH_2_PO_4_, 1 mM EDTA, 1% polyoxyethylene-10-tridecyl-ether, pH 7.4). The triacylglycerol concentration was measured with the Randox TR210 kit (Randox, Wülfrath, Germany) according to the manufacturer’s instructions.

### Microarray

Liver RNA was purified from four animals each (8 weeks) from B6-WT and B6-Tg(*Zfp69*) on SD and used for microarray analysis (Agilent Whole Genome Mouse 4 × 44K arrays, Source Bioscience, Berlin, Germany).

## Results

### C57BL/6J transgenic mice overexpressing *Zfp69*

In order to investigate the impact of *Zfp69* on glucose homeostasis and fat distribution, its myc-tagged cDNA fused to the ubiquitin C promoter was integrated into the ROSA locus of B6 genome (ESM Fig. 1a). At 8 weeks of age, *Zfp69* expression levels were examined in various tissues of the transgenic mouse line. As anticipated, *Zfp69* was markedly overexpressed in all tissues of the transgenic mice (ESM Fig. 1b), whereas mRNA levels were below the detection level (C_t_ value <35 by qPCR) in B6-WT. This increase in *Zfp69* mRNA levels gave rise to protein levels in liver nuclei that were twofold higher in *Zfp69* transgenic mice than in SJL (ESM Fig. 1c).

The *Zfp69* transgenic mice developed normally and did not show any alteration in body weight increment as compared with B6-WT mice (ESM Fig. 2).

### Mild insulin resistance in B6-Tg(*Zfp69*) mice

**Fig. 1:**
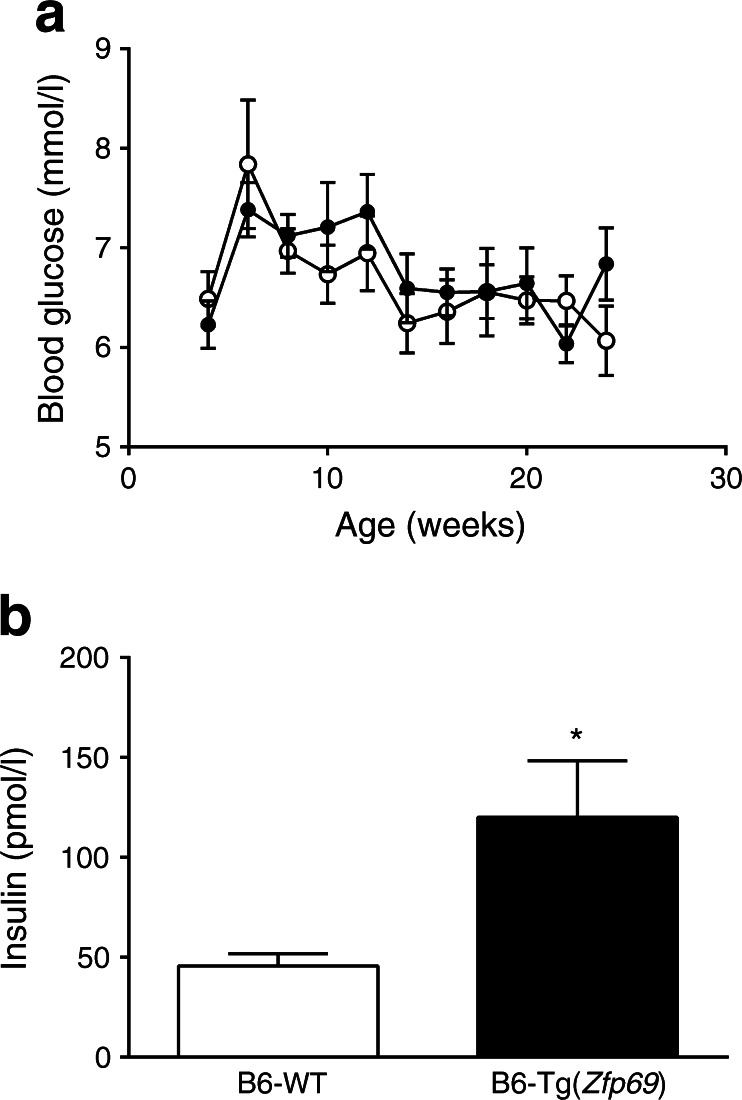
Increased plasma insulin levels in B6-Tg(*Zfp69*) mice. (**a**) Samples for measurement of randomly fed blood glucose levels were collected in the morning from mice on SD. White circles, B6-WT; black circles, B6-Tg(*Zfp69*). (**b**) Plasma insulin levels were measured in the postprandial state at the age of 24 weeks. Data are presented as mean ± SE of 7–11 mice. *p < 0.05 by Student’s *t* test

To examine the effect of *Zfp69* on glucose metabolism, blood glucose and insulin levels were compared between control and transgenic mice. *Zfp69* overexpression did not result in increased blood glucose levels in the fed status at any time point (Fig. 1a).

**Fig. 2:**
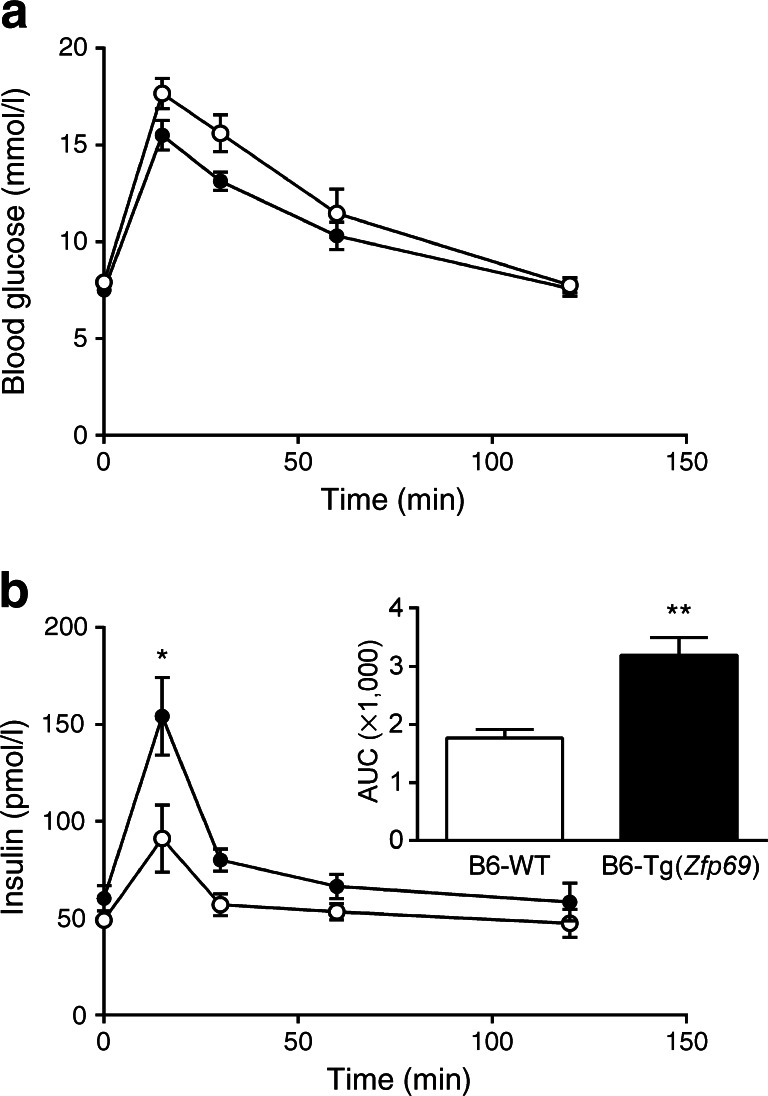
Increased insulin levels during OGTT in B6-Tg(*Zfp69*) mice. Mice kept on SD until 18 weeks of age were fasted for 6 h before oral glucose gavage (2 g/kg body weight). (**a**) Blood glucose and (**b**) corresponding insulin levels were measured at indicated time points. Area under the curve (AUC) values of insulin are depicted in the small panel in (**b**) (pmol/l × min). White circles, B6-WT; black circles, B6-Tg(*Zfp69*). Data are presented as mean ± SE of 10–11 animals. *p < 0.05, **p < 0.01 by Student’s *t* test

However, plasma insulin levels at 24 weeks of age were significantly higher in B6-Tg(*Zfp69*) than in WT mice (Fig. 1b), whereas total pancreatic insulin content did not differ between the groups (ESM Fig. 3a). Beta cell area in the pancreas did not show any difference between the genotypes (ESM Fig. 3b). Furthermore, glucose and glucose plus palmitate stimulation of isolated islets, as well as potassium chloride treatment, increased the secretion of insulin from both B6-WT and B6-Tg(*Zfp69*) islets (ESM Fig. 3c) without significant differences between genotypes. Glucose clearance during an OGTT at week 18 was not different between B6-WT and B6-Tg(*Zfp69*) mice (Fig. 2a), but the insulin concentration was significantly increased in B6-Tg(*Zfp69*) mice (Fig. 2b), suggesting that *Zfp69* overexpression causes mild insulin resistance. Additionally, in intraperitoneal GTT (IP-GTT) blood glucose levels were not different between B6-WT and B6-Tg(*Zfp69*) mice (ESM Fig. 4a). However, insulin levels during IP-GTT showed only a tendency to increase (ESM Fig. 4b).

Consistent with the assumption of a hepatic insulin resistance, the expression of proteins involved in glucose homeostasis, such as glucose-6-phosphatase (encoded by *G6pc*) and phosphoenolpyruvate carboxykinase1 (encoded by *Pck1*), was significantly increased in livers of B6-Tg(*Zfp69*) mice at week 24 (ESM Fig. 5).

### Effect of *Zfp69* on glucose metabolism in obese mouse models

Since the diabetogenic effect of the *Zfp69* locus required obesity, we challenged control and B6-Tg(*Zfp69*) mice with a HFD, which markedly increased body weight. In addition, NZO mice were crossed with B6-Tg(*Zfp69*) mice to produce obese NZO/B6 F1 progeny overexpressing *Zfp69*. Like B6-Tg(*Zfp69*) mice, NZO/B6-Tg(*Zfp69*) mice overexpressed *Zfp69* in fat tissues, muscle and liver (ESM Fig. 6a). Blood glucose in the fed status did not differ between NZO/B6-WT and NZO/B6-Tg(*Zfp69*) mice (ESM Fig. 6b). As expected, the body weight of NZO/B6 F1 mice was twofold higher than that of B6 mice, with no differences between genotypes (ESM Fig. 6c), suggesting that *Zfp69* overexpression does not alter growth or fat accumulation.

**Fig. 3:**
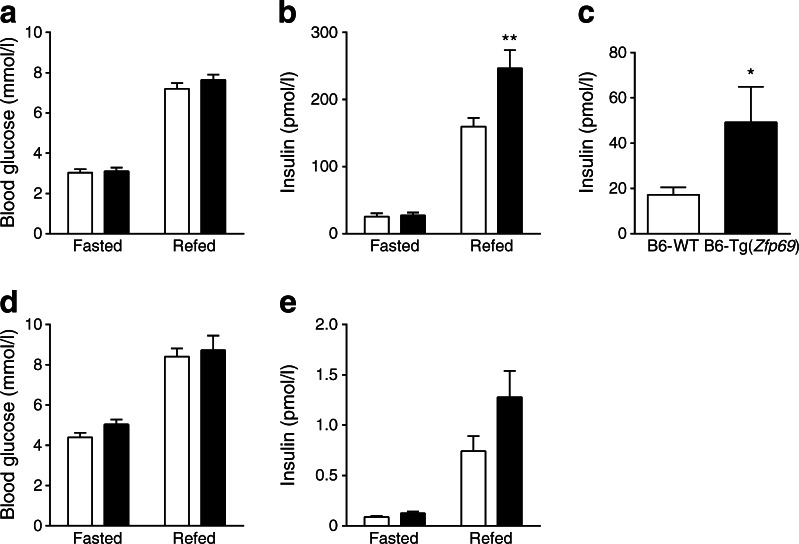
Increased postprandial insulin levels in obese mouse models expressing *Zfp69*. (**a**) Blood glucose and (**b**) corresponding insulin levels of 8-week-old B6-WT and B6-Tg(*Zfp69*) mice were measured after an overnight fast and 2 h after refeeding. (**c**) Insulin levels in 16 h fasted status of B6-WT and B6-Tg(*Zfp69*) mice fed on an HFD at 18 weeks of age. (**d**) Blood glucose and (**e**) corresponding insulin levels measured in 11-week-old NZO/B6-WT and NZO/B6-Tg(*Zfp69*) mice after an overnight fast and 2 h refeeding. Mice were kept on HFD until the experiment. White bars, WT mice; black bars, transgenic mice. Data are presented as mean ± SE of 9–11 animals. *p < 0.05, **p < 0.01 by two-way ANOVA with Bonferroni’s multiple comparisons test

In order to investigate whether or not *Zfp69* expression causes any alteration in glucose homeostasis, blood glucose levels and the corresponding insulin levels were measured after an overnight fast and 2 h after refeeding. Blood glucose levels did not differ between the genotypes in either condition (Fig. 3a), but insulin levels were significantly higher in the postprandial state of 8-week-old B6-Tg(*Zfp69*) mice on HFD (Fig. 3b). Interestingly, in week 18, B6-Tg(*Zfp69*) mice showed significantly higher fasted insulin concentrations than B6-WT mice (Fig. 3c), but with no changes in the corresponding blood glucose levels (data not shown). Similarly, NZO/B6-Tg(*Zfp69*) mice exhibited a tendency towards higher insulin levels after refeeding (Fig. 3e), whereas the blood glucose levels were not different between the genotypes (Fig. 3d).

In OGTT of B6-Tg(*Zfp69*) mice on HFD (week 22) blood glucose levels were not different (ESM Fig. 7a), whereas corresponding insulin levels in *Zfp69* transgenic mice tended to be increased (ESM Fig. 7b). ITTs revealed a tendency towards increased blood glucose levels in B6-Tg(*Zfp69*) mice on a HFD (ESM Fig. 8b) compared with controls, indicating impaired insulin sensitivity.

### *Zfp69* overexpression increases liver fat and plasma triacylglycerol levels and decreases pAKT

**Fig. 4:**
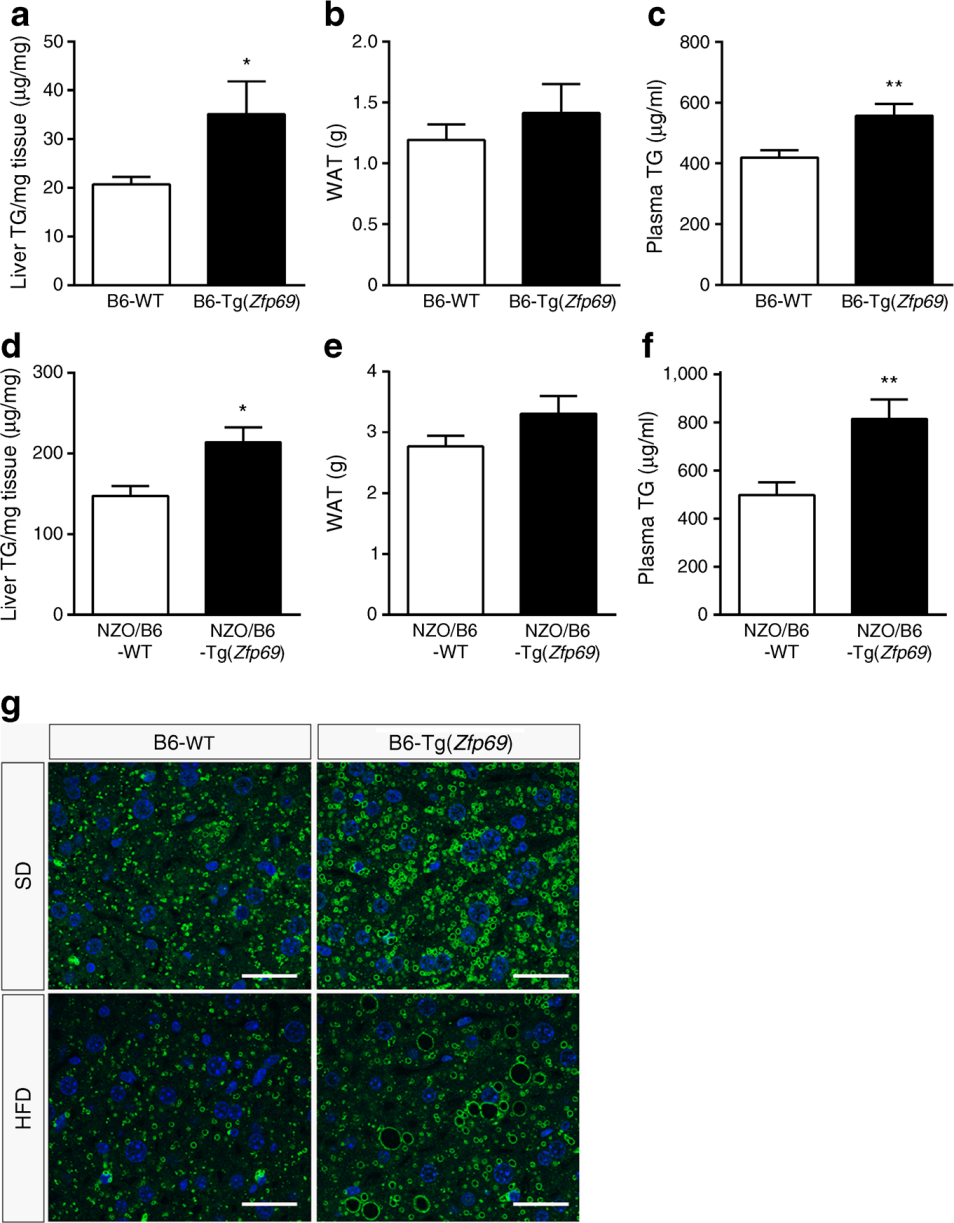
Increase in liver fat and plasma triacylglycerol (TG) levels in obese mice expressing *Zfp69*. B6-WT and transgenic mice fed with HFD were killed at week 24 at postprandial status. (**a**) Liver triacylglycerol levels and (**b**) gonadal fat mass (white adipose tissue [WAT]) was determined. (**c**) Plasma triacylglycerol levels were measured at a postprandial state in B6 background mice at 12 weeks of age. NZO/B6 mice (20 weeks old) fed on an HFD were killed at postprandial status. (**d**) Liver triacylglycerol levels and (**e**) gonadal fat mass were determined. (**f**) Plasma triacylglycerol levels of NZO/B6 mice were estimated at postprandial status at week 23. Data are presented as mean ± SE of 7–9 animals. *p < 0.05, **p < 0.01 by Student’s *t* test. (**g**) Plin2 staining of the liver sections of B6-WT and B6-Tg(*Zfp69*). Livers were taken after 6 h fasting from mice on SD (20 weeks) and HFD (22 weeks). Scale bar, 30 μm

Lean mice receiving a HFD as well as obese mice often develop hepatosteatosis which participates in the development of insulin resistance [16]. To test whether or not *Zfp69* overexpression enhances hepatic fat storage, we measured the liver triacylglycerol concentrations and detected significantly elevated levels, in both B6-Tg(*Zfp69*) and NZO/B6-Tg(*Zfp69*) mice on HFD (Fig. 4a and d); on SD this effect did not reach statistical significance (ESM Fig. 9). Lipid droplets, visualised by Plin2 staining were greater in numbers and larger in size in livers of B6-Tg(*Zfp69*) mice on both SD and HFD compared with WT mice (Fig. 4g). Hepatosteatosis was not due to hyperphagia because food intake did not differ between control and B6-Tg(*Zfp69*) mice on HFD (ESM Fig. 10).

The mass of the white gonadal fat pad was not affected by *Zfp69* overexpression in these mice (Fig. 4b and e). However, plasma triacylglycerol levels were significantly higher in *Zfp69* transgenic mice on B6 and NZO/B6 background (Fig. 4c and f) compared with controls, as observed in the *Zfp69* overexpressing congenic mice in a previous study [11]. Furthermore, we evaluated the fat distribution of B6-WT and B6-Tg(*Zfp69*) mice at 8 and 16 weeks of age using CT but did not detect any difference (data not shown).

**Fig. 5:**
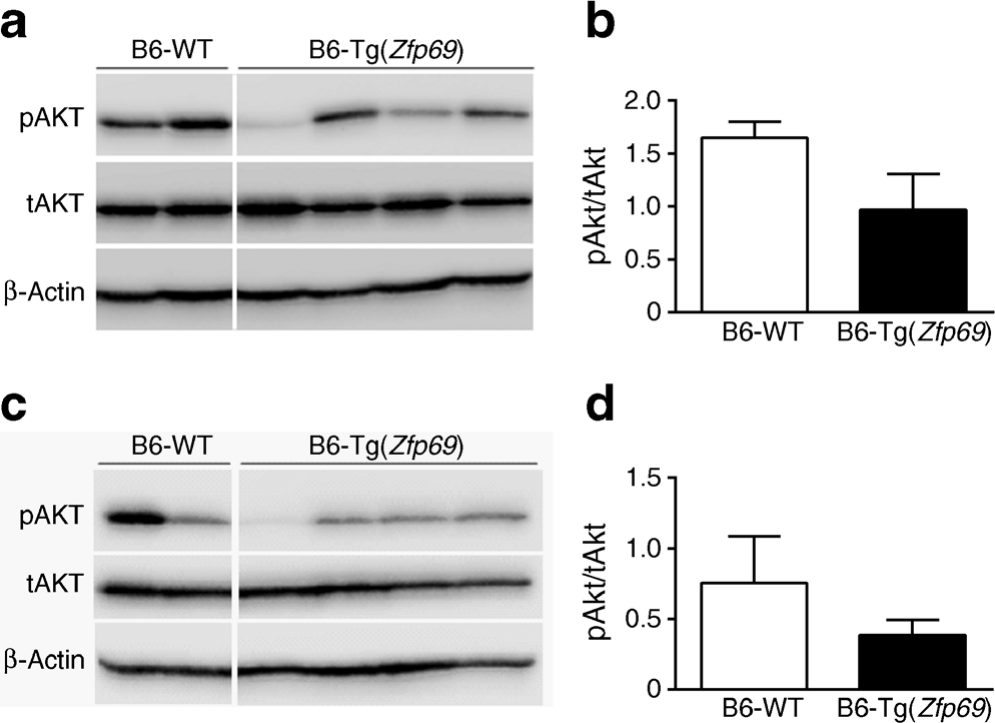
Slightly reduced hepatic pAKT levels in B6-Tg(*Zfp69*) mice. B6 mice on SD (20 weeks) were killed 20 min after an intraperitoneal injection of insulin (1 U). Western blots of pAKT and tAKT in the liver of B6-WT and B6-Tg(*Zfp69*) mice on (**a**) SD and (**c**) HFD. Quantification of pAKT/tAKT ratio of (**b**) SD and (**d**) HFD fed mice. β-actin was determined as a loading control. Data are presented as mean ± SE of two to four animals

In order to examine whether or not *Zfp69* overexpression affects hepatic insulin sensitivity, we investigated levels of pAKT in livers of B6 mice 20 min after intraperitoneal insulin application. We detected a tendency towards decreased pAKT levels in B6-Tg(*Zfp69*) on SD and HFD compared with B6-WT livers (Fig. 5).

### *Zfp69* suppresses the expression of genes involved in glucose and lipid metabolism

In order to further investigate the effects of *Zfp69* overexpression on the molecular regulation of glucose and lipid metabolism in the liver, we compared the liver transcriptome of 8-week-old B6-WT and B6-Tg(*Zfp69*) mice by a microarray analysis. Overall, 76 genes met the predefined criteria for greater than 1.5-fold differential expression (signal intensity on microarray >90, *p* < 0.05; Student’s *t test* of four analyses per group). In livers from B6-Tg(*Zfp69*) mice, 20 genes were upregulated and 56 genes were downregulated (Tables 1 and 2).

**Table 1:** Upregulated genes in the liver of B6-Tg(*Zfp69*) mice

Gene bank ID	Gene symbol	Description	Ratio
NM_007919	*Cela2a*	Chymotrypsin-like elastase family, member 2A	6.55
NM_025350	*Cpa1*	Carboxypeptidase A1	6.00
NM_025583	*Ctrb1*	Chymotrypsinogen B1	5.55
NM_001033875	*Ctrc*	Chymotrypsin C (caldecrin)	5.52
NM_025469	*Clps*	Colipase, pancreatic	5.23
NM_019738	*Nupr1*	Nuclear protein 1	3.99
NM_007446	*Amy1*	Amylase 1, salivary	2.46
NM_024440	*Derl3*	Der1-like domain family, member 3	2.21
NM_177388	*Slc41a2*	Solute carrier family 41, member 2	2.13
NM_013769	*Tjp3*	Tight junction protein 3	1.79
NM_175138	*Dnaic1*	Dynein, axonemal, intermediate chain 1	1.78
NM_138302	*Ecgf1* (also known as *Tymp*)	Endothelial cell growth factor 1 (platelet-derived)	1.66
NM_146116	*Tubb2c* (also know as *Tubb4b*)	Tubulin, beta 2c	1.65
NM_009777	*C1qb*	Complement component 1, q subcomponent, beta polypeptide	1.57
NM_013612	*Slc11a1*	Solute carrier family 11 (proton-coupled divalent metal ion transporters), member 1	1.57
NM_007572	*C1qa*	Complement component 1, q subcomponent, alpha polypeptide	1.55
NM_017372	*Lyzs* (also known as *Lyz2*)	Lysozyme	1.52
NM_153074	*Lrrc25*	Leucine rich repeat containing 25	1.51
NM_001037859	*Csf1r*	Colony stimulating factor 1 receptor	1.51
NM_013632	*Pnp*	Purine-nucleoside phosphorylase	1.51

Livers were collected from mice on SD at 8 weeks of age

Genes with a greater than 1.5-fold increased expression are listed

**Table 2:** Downregulated genes in the liver of B6-Tg(*Zfp69*) mice

Gene bank ID	Gene symbol	Description	Ratio
NM_201256	*Eif4ebp3*	Eukaryotic translation initiation factor 4E binding protein 3	0.42
NM_032002	*Nrg4*	Neuregulin 4	0.45
NM_177368	*Tmtc2*	Transmembrane and tetratricopeptide repeat containing 2	0.50
NM_008898	*Por*	P450 (cytochrome) oxidoreductase	0.52
NM_013631	*Pklr*	Pyruvate kinase liver and red blood cell	0.55^a^
NM_207665	*Olfr144* (also known as *Olfr1537*)	Olfactory receptor 144	0.55
NM_021524	*Nampt*	Nicotinamide phosphoribosyltransferase	0.56^b^
NM_007812	*Cyp2a5*	Cytochrome P450, family 2, subfamily a, polypeptide 5	0.56
NM_011943	*Map2k6*	Mitogen-activated protein kinase 6	0.57^c^
NM_145572	*Gys2*	Glycogen synthase 2	0.59^d^
NM_009760	*Bnip3*	BCL2/adenovirus E1B interacting protein 1, NIP3	0.60^e^
NM_011391	*Slc16a7*	Solute carrier family 16 (monocarboxylic acid transporters), member 7	0.61
NM_172668	*Lrp4*	Low density lipoprotein receptor-related protein 4	0.63
NM_010361	*Gstt2*	Glutathione S-transferase, theta 2	0.64
NM_173397	*Fitm2*	Fat storage-inducing transmembrane protein 2	0.65^f^
NM_009647	*Ak3l1* (also known as *Ak4*)	Adenylate kinase 3 alpha-like 1	0.65
NM_007618	*Serpina6*	Serine (or cysteine) peptidase inhibitor, clade A, member 6	0.65
NM_007520	*Bach1*	BTB and CNC homology 1	0.66
NM_172838	*Slc16a12*	Solute carrier family 16 (monocarboxylic acid transporters), member 12	0.66
NM_031884	*Abcg5*	ATP-binding cassette, sub-family G (WHITE), member 5	0.67
NM_008772	*P2ry1*	Purinergic receptor P2Y, G-protein coupled 1	0.68
NM_172563	*Hlf*	Hepatic leukemia factor	0.68
NM_001081131	*Dhtkd1*	Dehydrogenase E1 and transketolase domain containing 1	0.68
NM_022882	*Lpin2*	Lipin 2	0.68^g^
NM_009030	*Rbbp4*	Retinoblastoma binding protein 4	0.69
NM_026003	*Smarca2*	SWI/SNF related, matrix associated, actin dependent regulator of chromatin, subfamily a, member 2	0.69
NM_024198	*Gpx7*	Glutathione peroxidase 7	0.69
NM_008813	*Enpp1*	Ectonucleotide pyrophosphatase/phosphodiesterase 1	0.70
NM_145076	*Trim24*	Tripartite motif protein 24	0.71
NM_024289	*Osbpl5*	Oxysterol binding protein-like 5	0.71
NM_031197	*Slc2a2*	Solute carrier family 2 (facilitated glucose transporter), member 2	0.71^h^
NM_008188	*Thumpd3*	THUMP domain containing 3	0.71
NM_001081260	*Tnks1bp1*	Tankyrase 1 binding protein 1	0.71
NM_020567	*Gmnn*	Geminin	0.72
NM_028790	*Acot12*	Acyl-CoA thioesterase 12	0.72
NM_008904	*Ppargc1a*	Peroxisome proliferative activated receptor, gamma, coactivator 1 alpha	0.72^i^
NM_022722	*Dpys*	Dihydropyrimidinase	0.72
NM_183262	*Stk35*	Serine/threonine kinase 35	0.72
NM_146078	*Ubr2*	Ubiquitin protein ligase E3 component n-recognin 2	0.73
NM_011200	*Ptp4a1*	Protein tyrosine phosphatase 4a1	0.73
NM_198300	*Cpeb3*	Cytoplasmic polyadenylation element binding protein 3	0.73
NM_009981	*Pcyt1a*	Phosphate cytidylyltransferase 1, choline, alpha isoform	0.73
NM_009738	*Bche*	Butyrylcholinesterase	0.74
NM_178378	*Iqcg*	IQ motif containing G	0.74
NM_011864	*Papss2*	3′-phosphoadenosine 5′-phosphosulfate synthase 2	0.74
NM_177327	*Wwp1*	WW domain containing E3 ubiquitin protein ligase 1	0.74
NM_172907	*Olfml1*	Olfactomedin-like 1	0.74
NM_145823	*Pitpnc1*	Phosphatidylinositol transfer protein, cytoplasmic 1	0.74
NM_010568	*Insr*	Insulin receptor	0.74^j^
NM_013505	*Dsc2*	Desmocollin 2	0.74
NM_001013391	*Cpsf6*	Cleavage and polyadenylation specific factor 6	0.74
NM_011387	*Slc10a1*	Solute carrier family 10 (sodium/bile acid cotransporter family), member 1	0.74
NM_007996	*Fdx1*	Ferredoxin 1	0.74
NM_177321	*Mia2*	Melanoma inhibitory activity 2	0.74
NM_022996	*Ndfip1*	Nedd4 family interacting protein 1	0.75
NM_153599	*Cdk8*	Cyclin-dependent kinase 8	0.75

Livers were collected from mice on SD at 8 weeks of age

Genes with reduced expression by >25% are listed

^a–j^The ten genes selected by a literature survey and a MetaCore-based pathway enrichment analysis as being related to diabetes and lipid metabolism

**Fig. 6:**
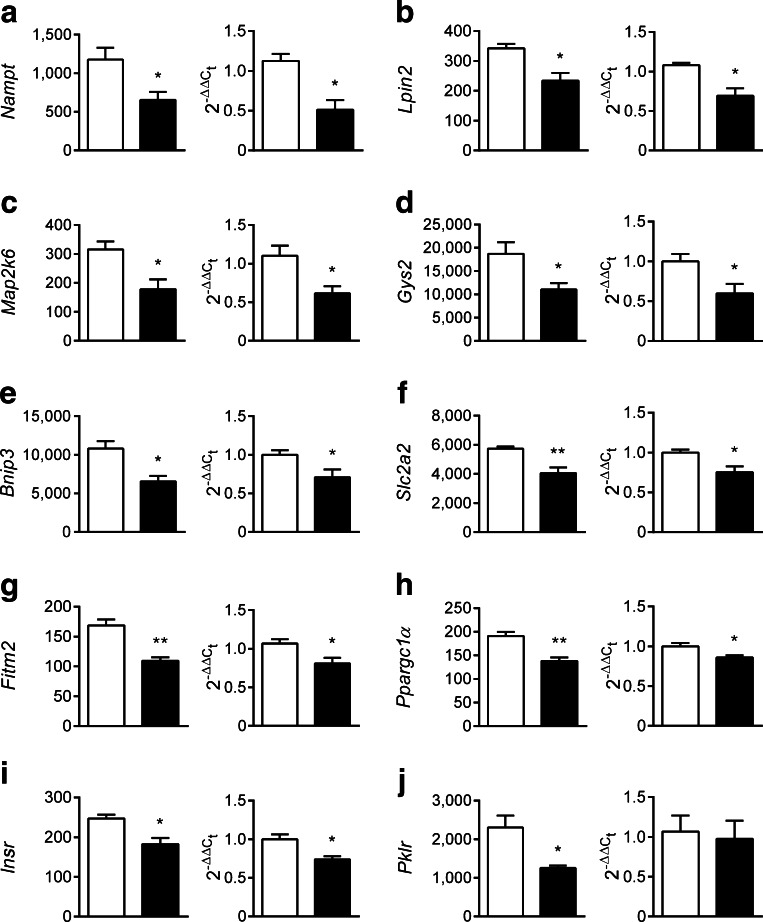
Suppression of hepatic genes involved in glucose and lipid metabolism in response to *Zfp69* overexpression. Genes involved in glucose and lipid metabolism were selected by MetaCore-based pathway enrichment analysis among genes exhibiting >25% decreased expression in the liver of B6-Tg(*Zfp69*) mice at 8 weeks of age on SD. (**a**) *Nampt* (**b**) *Lpin2* (**c**) *Map2k6* (**d**) *Gys2* (**e**) *Bnip3* (**f**) *Slc2a2* (**g**) *Fitm2* (**h**) *Ppargc1α* (**i**) *Insr* and (**j**) *Pklr.* Each gene is presented by the signal intensity on microarray (left graph) and the expression validation by qPCR (right graph). White bars, B6-WT; black bars, B6-Tg(*Zfp69*). Data are presented as mean ± SE of three to four animals. *p < 0.05, **p < 0.01 by Student’s *t* test

Since ZFP69 is an inhibitory regulator of gene expression by analogy, we further analysed the genes that were suppressed in livers of B6-Tg(*Zfp69*) mice. According to a literature survey and a MetaCore-based pathway enrichment analysis, ten genes with a reduced expression by >25% were related to diabetes and lipid metabolism (Table 2). The *Zfp69*-dependent suppression of nine of these genes, *Nampt, Lpin2, Map2k6, Gys2, Bnip3, Fitm2*, *Slc2a2*, *Ppargc1α* and *Insr* was validated by qPCR (Fig. 6).

## Discussion

*Zfp69* has been previously suggested to be a causal gene in the diabetes loci *Nidd1* and *Nidd/SJL* [11]. In this study, we present direct evidence in support of this conclusion, and demonstrate that *Zfp69* increases liver fat content and plasma triacylglycerol concentrations and causes moderate insulin resistance.

B6-Tg(*Zfp69*) mice displayed elevated insulin levels in OGTTs at weeks 18 and 24 compared with B6-WT animals. Interestingly, IP-GTT did not significantly increase insulin levels. As IP-GTT by-passes incretin stimulation, in contrast to OGTT, it can be speculated that *Zfp69* overexpression increases incretin release and thereby insulin secretion. Along these lines, we did not detect an elevated glucose-stimulated insulin secretion in isolated islets of transgenic mice.

Elevated liver triacylglycerol concentrations, slightly reduced hepatic pAKT levels and higher glucose levels during ITT of HFD fed mice indicate that *Zpf69* mediates a moderate insulin resistance. Accordingly, hepatic expression of *G6pc* and *Pck1* was significantly increased, as shown by others in the insulin-resistant state [17, 18]. The higher liver fat and plasma triacylglycerol levels could result either from hepatic insulin resistance or from *Zfp69* overexpression in adipose tissue. Similarly, in a previous study, we observed hepatosteatosis and hyperlipidaemia in recombinant congenic mice carrying the *Zfp69* locus of SJL mice and hypothesised that this is due to reduced lipid storage capacity of adipocytes [11]. However, as *Zfp69* transgenic mice did not exhibit smaller fat pads, elevated plasma triacylglycerol concentrations appear not to be a consequence of elevated lipolysis.

Overall, the phenotype of the transgenic mice is much weaker than that of recombinant congenic mice carrying the *Zfp69* locus of SJL mice [11]. Contrary to our expectations, we did not observe hyperglycaemia or beta cell failure in B6-Tg(*Zfp69*) or in NZO/B6-Tg(*Zfp69*) mice. A possible explanation for the mild phenotype could be that *Zfp69* needs other diabetogenic gene variants in order to produce hyperglycaemia and beta cell failure. We have previously shown that the introgression of *Nidd/SJL* encompassing the intact *Zfp69* into the NZO background induced hypoinsulinaemia due to beta cell failure, resulting in hyperglycaemia [9]. However, when the *Nidd/SJL* locus was introgressed into B6-*ob* mice, it caused an altered fat distribution (hepatosteatosis and a reduced white gonadal fat depot) as well as mild hyperglycaemia, but not beta cell failure [11], consistent with the assumption that B6 mice carry diabetes suppressing genes [6]. Moreover, previous studies showed that *Nidd/SJL* interacts with NZO genes (e.g. on chromosomes 1 and 15) that enhance the diabetogenic effect of *Nidd/SJL* [9, 11].

In addition, the possibility that the *Nidd/SJL* locus harbours additional diabetogenic genes that are in linkage disequilibrium with *Zfp69* cannot be discounted. Indeed, a linkage study of an F2 intercross between B6 and DBA/2 mouse lines with a deficiency in the leptin receptor (*db/db*) identified an interval of chromosome 4 that was associated with the traits blood glucose and plasma triacylglycerols [19], confirming that the region is responsible for obesity-induced diabetes. Furthermore, from their genome-wide analysis on an (B6xC3H/HeJ)F2 intercross with a deficiency in apolipoprotein E, Logsdon et al concluded that *Zfp69* variants are associated with body weight, blood glucose and cholesterol levels [20].

We compared the liver transcriptome of B6-WT and B6-Tg(*Zfp69*) mice at an early time point (week 8) before insulin resistance occurs in order to exclude secondary effects of insulin resistance and to examine the direct effect of *Zfp69* overexpression. Microarray analysis revealed that *Zfp69* overexpression decreased the expression of several genes involved in glucose and lipid metabolism. Interestingly, none of the upregulated genes in the microarray was related to glucose or lipid metabolism.

*Nampt* plays a key role in NAD synthesis, which is needed for the enzymatic activity of sirtuin1 (SIRT1), an important regulator of glucose and lipid metabolism [21] and thereby regulates hepatic triacylglycerol homeostasis [22]. Decreased expression of *Nampt* could participate in the development of hepatic steatosis and insulin resistance by reducing SIRT1 activity. Hepatic deletion of SIRT1 impaired peroxisome proliferator-activated receptor α function, decreased fatty acid beta-oxidation and caused hepatic steatosis [23].

*Lpin2* is one of three members of the lipin family that act as phosphatidate phosphatases. These enzymes are required for glycerolipid biosynthesis and also act as transcriptional co-activators that regulate expression of lipid metabolising genes. Interestingly, polymorphisms in the *LPIN1* and *LPIN2* genes are associated with traits of metabolic disease, including insulin sensitivity, diabetes and increased blood pressure, as well as the response to thiazolidinediones [24]. Furthermore, *Lpin1* expression levels in adipose tissue and liver are positively correlated with insulin sensitivity [25]. Thus, a reduced *Lpin2* expression in the liver of B6-Tg(*Zfp69*) mice might affect lipid metabolism leading to insulin resistance.

*Gys2* catalyses the rate-limiting step in the synthesis of glycogen. The liver-specific deletion of *Gys2* resulted not only in a marked reduction of glycogen storage but also in impaired glucose tolerance [26]. Mitogen-activated protein kinase (MAPK) kinase 6 (MAP2K6) is a dual-specificity protein kinase that activates the stress-activated protein p38 MAPK [27]. In the livers of obese mice, p38 MAPK activity was markedly reduced compared with that of lean mice [28]. The liver-specific overexpression of constitutively active MAP2K6 (MKK6Glu) in obese *ob/ob* mice markedly reduced plasma insulin concentrations and improved glucose tolerance [28], indicating that reduced *Map2k6* expression as detected in B6-Tg(*Zfp69*) mice might impair glucose homeostasis.

Reduced *Bnip3* in mice overexpressing *Zfp69* can be also linked to elevated hepatic fat storage because deletion of *Bnip3* in mice resulted in increased lipid synthesis in the liver [29]. Bnip3 localises to the outer mitochondrial membrane and plays an important role in mitophagy and mitochondrial dynamics and is thereby vital in the adaptive response to changes in energy balance arising from deficiencies in oxygen or glucose availability [30].

*Fitm2* encodes the fat storage-inducing transmembrane protein2, an evolutionarily conserved protein that is directly involved in fat storage. It is located in the endoplasmic reticulum and involved in partition of triacylglycerol into lipid droplets [31]. In a three-stage association study performed with an east Asian population *FITM2* was shown to associate with type 2 diabetes [32].

*Ppargc1α* is a transcriptional coactivator that is a central inducer of mitochondrial biogenesis [33] and a regulator of gluconeogenesis [34, 35]. As increased hepatosteatosis was shown to correlate with reduced *Ppargc1α* expression [36], it can be speculated that lower *Ppargc1α* levels in the liver of B6-Tg(*Zfp69*) mice participate in ectopic fat storage and subsequently cause insulin resistance.

The molecular mechanism by which *Zfp69* regulates the expression of target genes remains to be elucidated and further studies are required to provide direct functional evidence that *Zfp69* modulates these candidates directly. By sequence homology, *Zfp69* is a member of KRAB domain zinc finger proteins, which are assumed to suppress expression of target genes [37]. Our microarray results reflect this to some extent, with considerably more genes downregulated in the liver than upregulated.

In conclusion, our analysis of the phenotype of transgenic mice overexpressing *Zfp69* indicates that *Zfp69* plays a role lipid metabolism, liver fat accumulation and presumably in incretin release. Thus, the data are consistent with the conclusion that *Zfp69* is a causal gene of the diabetes locus *Nidd/SJL*. However, the data also suggest that *Zfp69* is not the only factor mediating the diabetogenic effect of this locus.

## Electronic supplementary material

ESM Fig. 1 (PDF 138 kb)

ESM Fig. 2 (PDF 97.1 kb)

ESM Fig. 3 (PDF 244 kb)

ESM Fig. 4 (PDF 127 kb)

ESM Fig. 5 (PDF 120 kb)

ESM Fig. 6 (PDF 281 kb)

ESM Fig. 7 (PDF 122 kb)

ESM Fig. 8 (PDF 90.1 kb)

ESM Fig. 9 (PDF 58.5 kb)

ESM Fig. 10 (PDF 103 kb)

ESM Table 1 (PDF 127 kb)

## Abbreviations

CT: Computed tomography
HFD: High-fat diet
IP-GTT: Intraperitoneal GTT
ITT: Insulin tolerance test
KRAB: Krüppel-associated box
MAPK: Mitogen-activated protein kinase
MAP2K6: MAPK kinase 6
NON: Non-obese non-diabetic
NZO: New Zealand obese
qPCR: Quantitative real-time PCR
QTL: Quantitative trait locus
SD: Standard diet
SIRT1: Sirtuin1
SJL: Swiss Jim Lambert
WT: Wild-type
