# Supplementary material for: The diabetes gene Zfp69 modulates hepatic insulin sensitivity in mice
Source: Diabetologia. 2015 Aug 1;58(10):2403–13. doi: 10.1007/s00125-015-3703-8 (PMC4572078; doi:10.1007/s00125-015-3703-8)
Supplement: Supplementary file 1 — (PDF 138 kb) [file 125_2015_3703_MOESM1_ESM.pdf]

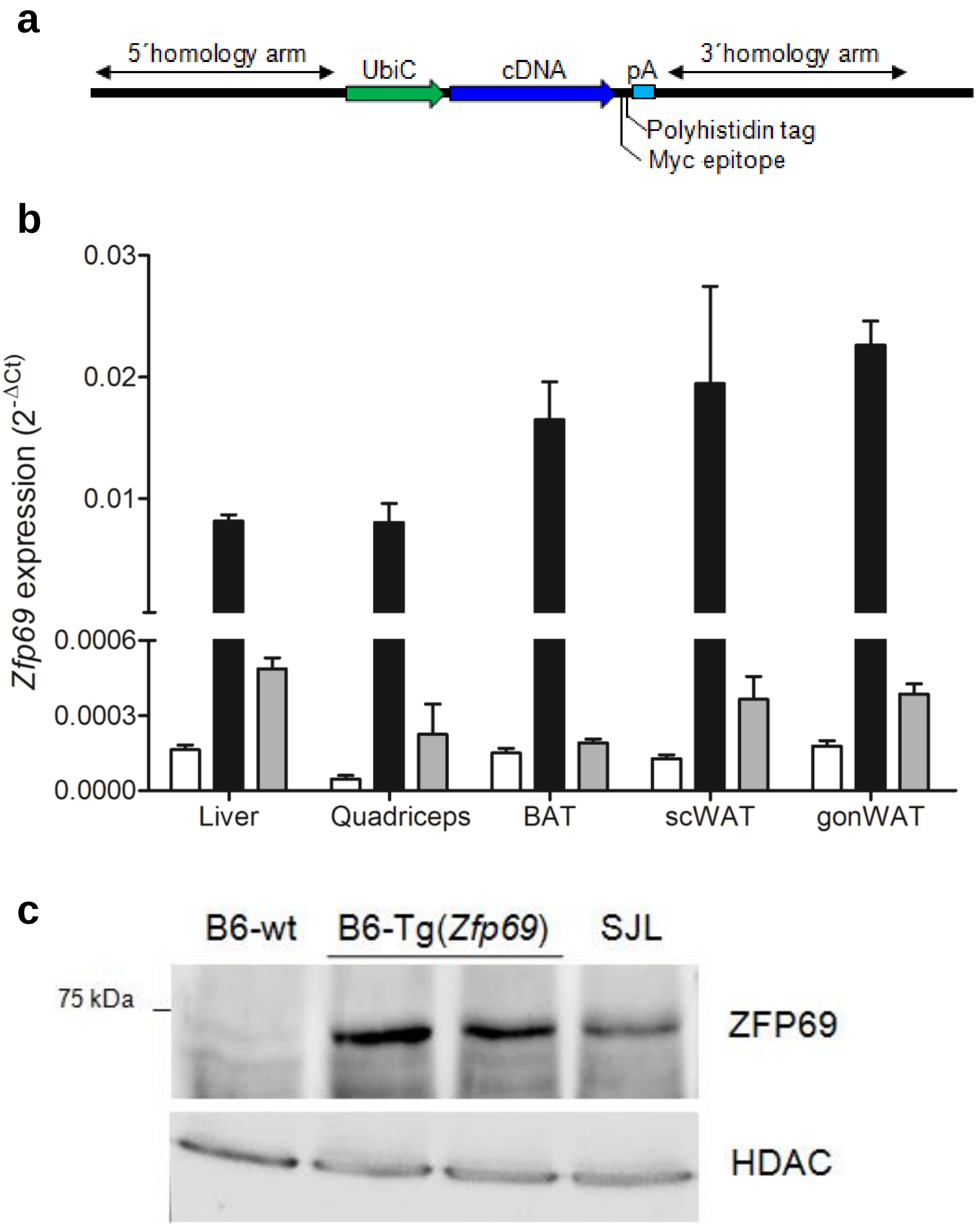

ESM Figure 1. Expression of *Zfp69* in B6-wt, B6-Tg(*Zfp69*) and SJL mice. (a) Structure of targeting vector integrated into the ROSA locus of B6 mice in order to overexpress *Zfp69*. *Zfp69* cDNA tagged with Myc epitope at the C-terminus was fused to the ubiquitin C promoter and flanked by sequences corresponding with the ROSA locus. (b) *Zfp69* mRNA levels in different tissues were detected by qPCR. Animals were fed a SD and were killed at 8 weeks of age. Expression of *Zfp69* was significantly higher in all tissues of B6-Tg(*Zfp69*) mice than B6-wt and SJL (one-way ANOVA with Tukey's *post hoc* test). White bars, B6-wt; black bars, B6-Tg(*Zfp69*); grey bars, SJL. (c) ZFP69 protein levels in the liver nuclear extracts were detected by western blot. HDAC was displayed as a loading control. Animals were fed a SD and were killed at 8 weeks of age.
