# Supplementary material for: The diabetes gene Zfp69 modulates hepatic insulin sensitivity in mice
Source: Diabetologia. 2015 Aug 1;58(10):2403–13. doi: 10.1007/s00125-015-3703-8 (PMC4572078; doi:10.1007/s00125-015-3703-8)
Supplement: Supplementary file 2 — (PDF 97.1 kb) [file 125_2015_3703_MOESM2_ESM.pdf]

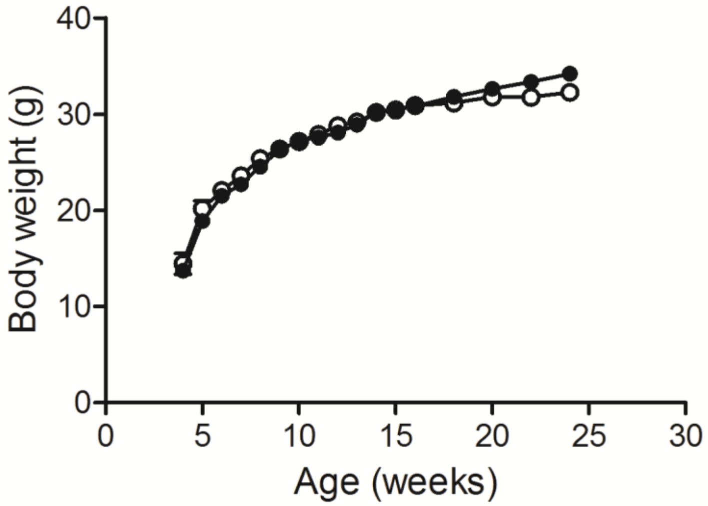

ESM Figure 2. Body weight development of B6-wt and B6-Tg(*Zfp69*) mice fed a standard diet. Data are presented as mean ± SE of 17 animals. White circles, B6-wt; black circles, B6-Tg(*Zfp69*)
