# Supplementary material for: The diabetes gene Zfp69 modulates hepatic insulin sensitivity in mice
Source: Diabetologia. 2015 Aug 1;58(10):2403–13. doi: 10.1007/s00125-015-3703-8 (PMC4572078; doi:10.1007/s00125-015-3703-8)
Supplement: Supplementary file 3 — (PDF 244 kb) [file 125_2015_3703_MOESM3_ESM.pdf]

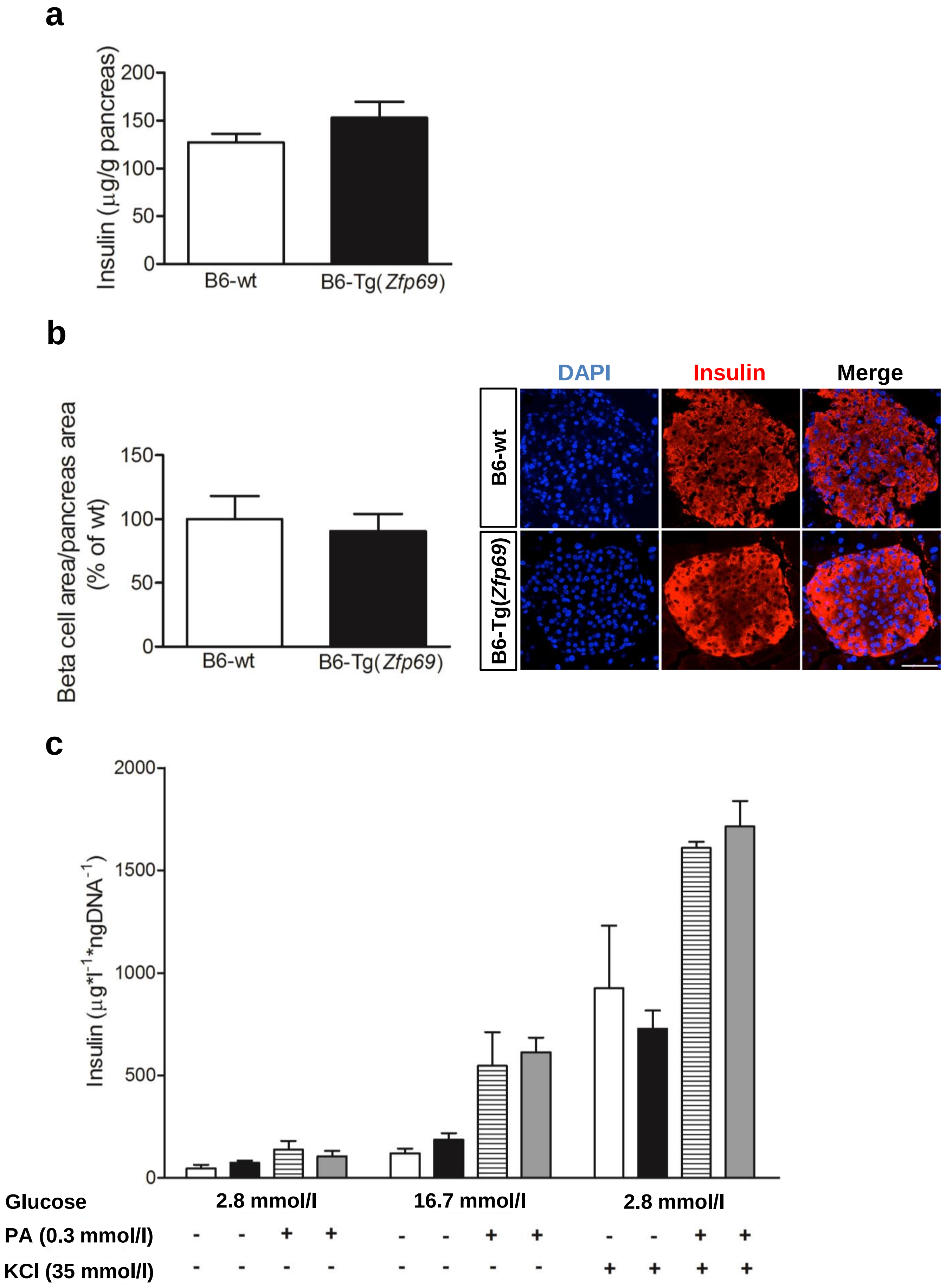
a
Insulin (μg/g pancreas)
200
150
100
50
0
B6-wt
B6-Tg(Zfp69)
b
Beta cell area/pancreas area (% of wt)
150
100
50
0
B6-wt
B6-Tg(Zfp69)
DAPI
Insulin
Merge
B6-wt
B6-Tg(Zfp69)
c
Insulin (μg*l-1*ngDNA-1)
2000
1500
1000
500
0
Glucose
2.8 mmol/l
16.7 mmol/l
2.8 mmol/l
PA (0.3 mmol/l)
- - + + - - + + - - + +
KCl (35 mmol/l)
- - - - - - - - + + + +

ESM Figure 3

**ESM Figure 3. *Zfp69* does not alter islet insulin content, histology or insulin secretion.** (a) Total pancreatic insulin was determined as described in Methods at 24 weeks of age. (b) Quantification of relative beta-cell area in the pancreas of B6-wt and B6-Tg(*Zfp69*) mice at 24 weeks of age on SD. Representative pictures are shown in the right panel. Data are presented as mean ± SE of 5 animals. (c) Pancreatic islets were isolated from B6-wt and B6-Tg(*Zfp69*) mice at 21 weeks of age. After incubation in 2.8 mmol/l glucose for 1 h, the islets were stimulated with 16.7 mmol/l glucose alone or plus 0.3 mmol/l palmitate. Insulin was measured in the supernatant of the islets after the stimulation. White bars, B6-wt –PA; black bars, B6-Tg(*Zfp69*) –PA; striped bars, B6-wt +PA; grey bars, B6-Tg(*Zfp69*) +PA
