# Supplementary material for: The diabetes gene Zfp69 modulates hepatic insulin sensitivity in mice
Source: Diabetologia. 2015 Aug 1;58(10):2403–13. doi: 10.1007/s00125-015-3703-8 (PMC4572078; doi:10.1007/s00125-015-3703-8)
Supplement: Supplementary file 4 — (PDF 127 kb) [file 125_2015_3703_MOESM4_ESM.pdf]

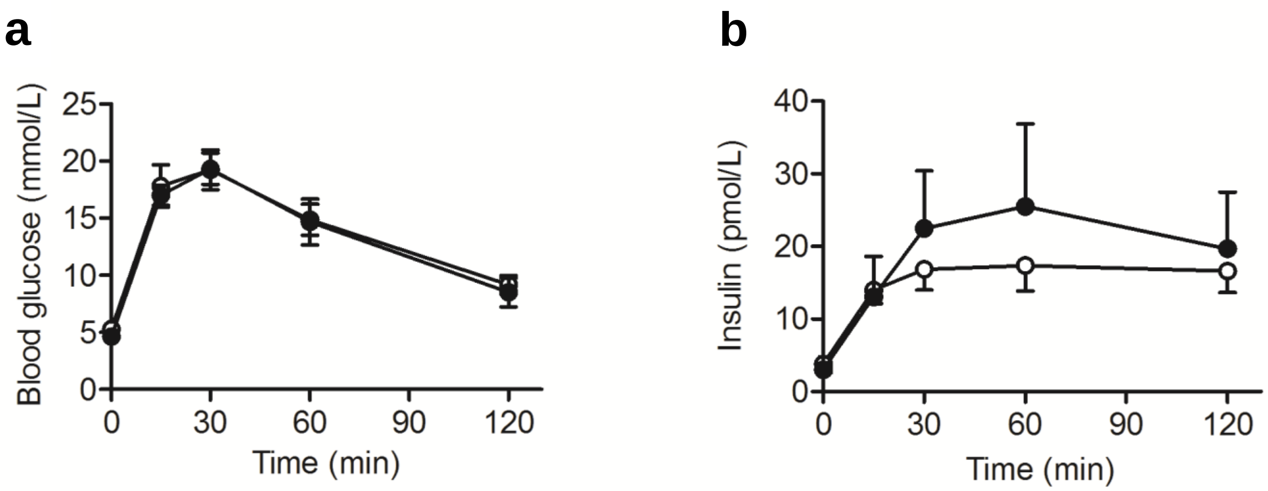

ESM Figure 4. Blood glucose and insulin levels during an IP-GTT in mice on SD. Mice were fasted overnight prior to glucose injection (2 g/kg body weight). Blood was taken to measure glucose and insulin levels at indicated time points. Development of blood glucose levels (a) and corresponding insulin levels (b) of 18 weeks old mice. White circles, B6-wt; black circles, B6-Tg(*Zfp69*). Data are presented mean ± SE of 4-6 animals.
