# Supplementary material for: The diabetes gene Zfp69 modulates hepatic insulin sensitivity in mice
Source: Diabetologia. 2015 Aug 1;58(10):2403–13. doi: 10.1007/s00125-015-3703-8 (PMC4572078; doi:10.1007/s00125-015-3703-8)
Supplement: Supplementary file 5 — (PDF 120 kb) [file 125_2015_3703_MOESM5_ESM.pdf]

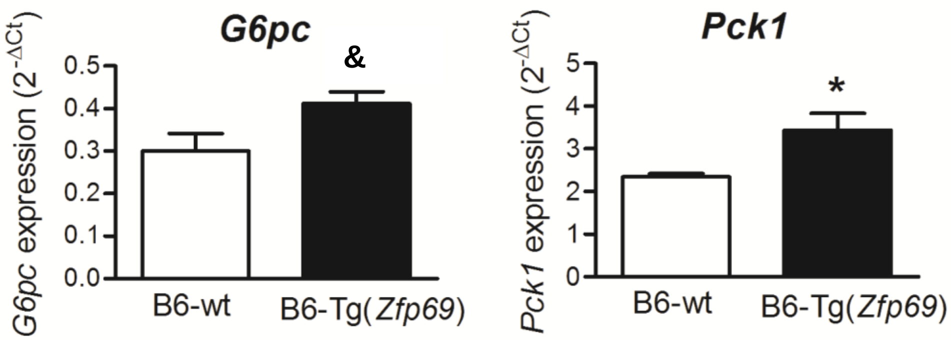

ESM Figure 5. Expression of *G6pc* and *Pck1* in the liver of B6-wt and B6-Tg(*Zfp69*) mice. Gene expression was determined by qPCR at 24 weeks of age in mice fed a SD. Data are presented as mean ± SE of 4-5 animals. $^{\&}p=0.06$ $^{*}p<0.05$; *t* test
