# Supplementary material for: The diabetes gene Zfp69 modulates hepatic insulin sensitivity in mice
Source: Diabetologia. 2015 Aug 1;58(10):2403–13. doi: 10.1007/s00125-015-3703-8 (PMC4572078; doi:10.1007/s00125-015-3703-8)
Supplement: Supplementary file 6 — (PDF 281 kb) [file 125_2015_3703_MOESM6_ESM.pdf]

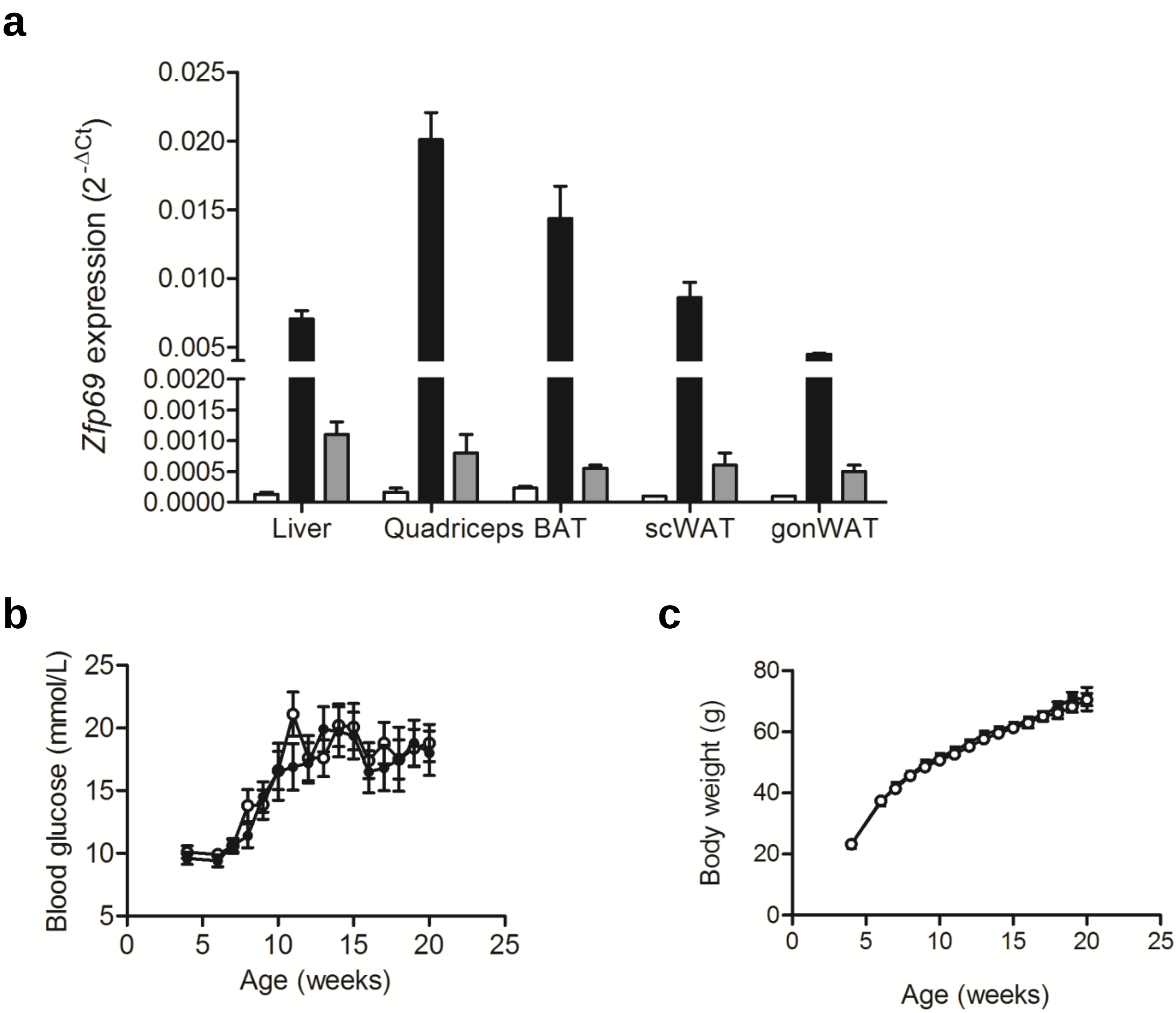

ESM Figure 6. *Zfp69* expression, development of hyperglycaemia and body weight in NZO/B6-Tg(*Zfp69*) mice. (a) Expression of *Zfp69* in different tissues of NZO/B6-wt and NZO/B6-Tg(*Zfp69*) mice as determined by qPCR. Animals were killed at 8 weeks of age. Data are presented as mean ± SE of 3 animals. Expression of *Zfp69* was significantly higher in all tissues of NZO/B6-Tg(*Zfp69*) mice than wt and SJL (one-way ANOVA with Tukey's *post hoc* test). White bars, NZO/B6-wt; black bars, NZO/B6-Tg(*Zfp69*); grey bars, SJL. (b) Time course of the development of hyperglycaemia in NZO/B6-wt and NZO/B6-Tg(*Zfp69*) mice. (c) Body weight development of NZO/B6-wt and NZO/B6-Tg(*Zfp69*) mice. All mice were fed a HFD. White circles, NZO/B6-wt; black circles, NZO/B6-Tg(*Zfp69*). Data are presented as mean ± SE of 16 animals.
