# Supplementary material for: The diabetes gene Zfp69 modulates hepatic insulin sensitivity in mice
Source: Diabetologia. 2015 Aug 1;58(10):2403–13. doi: 10.1007/s00125-015-3703-8 (PMC4572078; doi:10.1007/s00125-015-3703-8)
Supplement: Supplementary file 7 — (PDF 122 kb) [file 125_2015_3703_MOESM7_ESM.pdf]

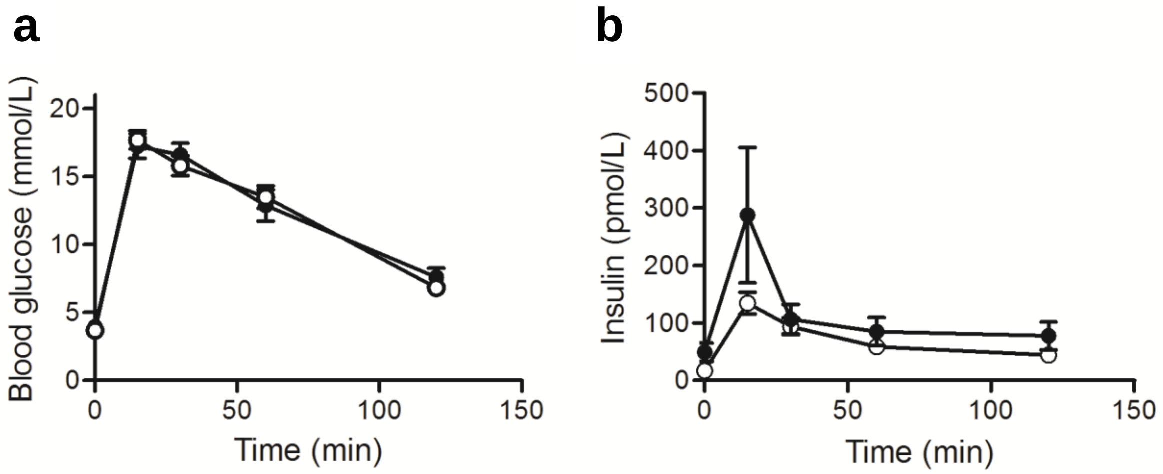

ESM Figure 7. Mild effect of *Zfp69* overexpression on insulin levels during OGTT. Mice on a HFD at 22 weeks of age were fasted for 16 h before oral glucose gavage (2 g/kg body weight). Blood glucose (a) and corresponding insulin levels (b) were measured at indicated time points. White circles, B6-wt; black circles, B6-Tg(*Zfp69*). Data are presented as mean ± SE of 5-7 animals.
