# Supplementary material for: The diabetes gene Zfp69 modulates hepatic insulin sensitivity in mice
Source: Diabetologia. 2015 Aug 1;58(10):2403–13. doi: 10.1007/s00125-015-3703-8 (PMC4572078; doi:10.1007/s00125-015-3703-8)
Supplement: Supplementary file 8 — (PDF 90.1 kb) [file 125_2015_3703_MOESM8_ESM.pdf]

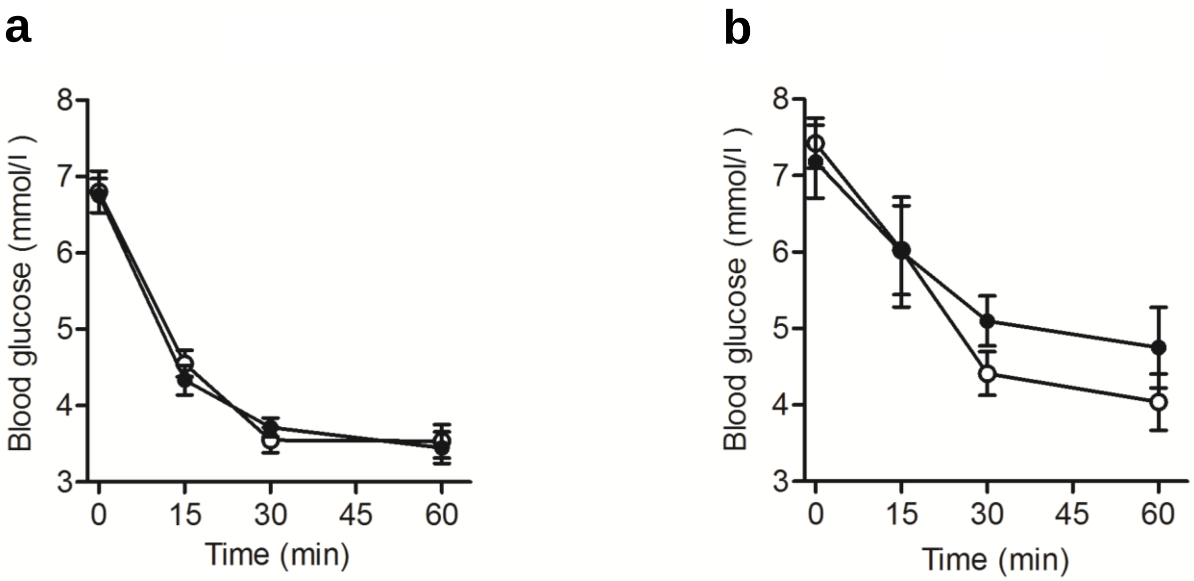

ESM Figure 8. Insulin tolerance test in B6-wt and B6-Tg(*Zfp69*) mice. Mice were intraperitoneally injected with insulin (1 IU for SD animals and 1.25 IU for HFD animals) and blood glucose levels were measured at indicated time points. (a) Blood glucose levels during insulin tolerance test of mice on SD at 12-15 weeks of age. Data are presented mean ± SE of 6 animals. (b) Blood glucose levels during insulin tolerance test of mice on HFD at 22 weeks of age. White circles, B6-wt; black circles, B6-Tg(*Zfp69*). Data are presented mean ± SE of 6-8 animals.
