# Supplementary material for: The diabetes gene Zfp69 modulates hepatic insulin sensitivity in mice
Source: Diabetologia. 2015 Aug 1;58(10):2403–13. doi: 10.1007/s00125-015-3703-8 (PMC4572078; doi:10.1007/s00125-015-3703-8)
Supplement: Supplementary file 9 — (PDF 58.5 kb) [file 125_2015_3703_MOESM9_ESM.pdf]

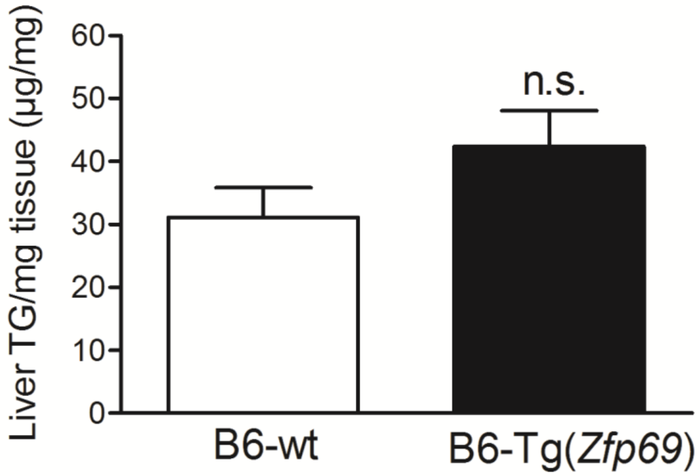

ESM Figure 9. Liver fat in B6-wt and B6-Tg(*Zfp69*) mice. Triacylglycerol levels were determined in livers of B6-wt and B6-Tg(*Zfp69*) mice fed a SD at 24 weeks of age. Data are presented mean ± SE of 6 animals. n.s.; not significant by *t* test
