# Supplementary material for: The diabetes gene Zfp69 modulates hepatic insulin sensitivity in mice
Source: Diabetologia. 2015 Aug 1;58(10):2403–13. doi: 10.1007/s00125-015-3703-8 (PMC4572078; doi:10.1007/s00125-015-3703-8)
Supplement: Supplementary file 10 — (PDF 103 kb) [file 125_2015_3703_MOESM10_ESM.pdf]

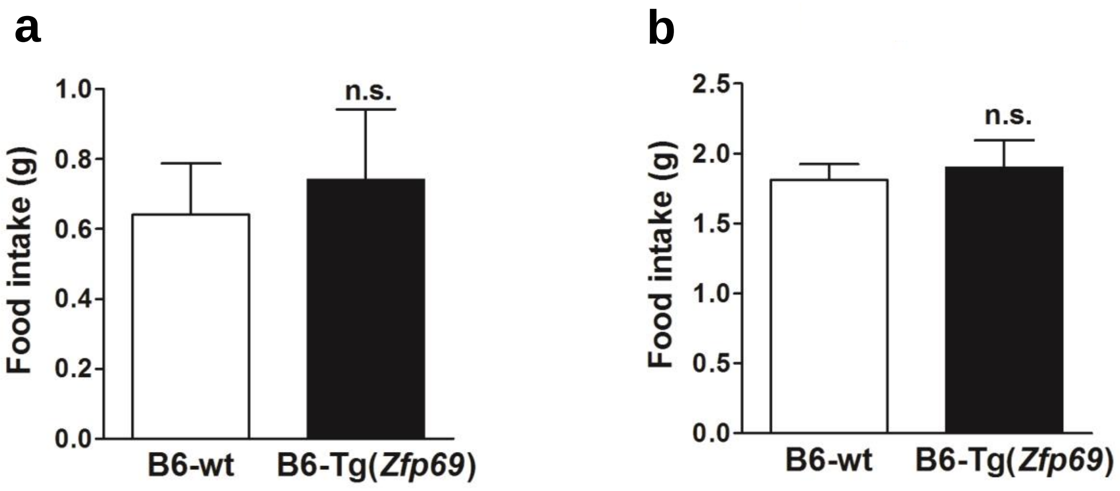

ESM Figure 10. Food intake of B6-wt and B6-Tg(*Zfp69*) mice. Food intake during the light (a) and dark phases (b) was measured in mice kept on HFD at 8 weeks of age (light on at 6 am and off at 6 pm). Data are presented as mean ± SE of 6 animals. n.s.; not significant by *t* test
