# Supplementary material for: The diabetes gene Zfp69 modulates hepatic insulin sensitivity in mice
Source: Diabetologia. 2015 Aug 1;58(10):2403–13. doi: 10.1007/s00125-015-3703-8 (PMC4572078; doi:10.1007/s00125-015-3703-8)
Supplement: Supplementary file 11 — (PDF 127 kb) [file 125_2015_3703_MOESM11_ESM.pdf]

Supplementary table 1. Primer sequences and Taqman assays that were used for qRT-PCR

Gene symbol	Forward primer<br>Reverse primer or Taqman assay	UPL probe no
Nampt	GGCAGAAGCCGAGTTCAA<br>TGGGTGGGTATTGTTTATAGTGAG	18
Lpin2	TCTGAATGATATTAAGAACTTGTTTGC<br>TGGAACTCCGACTTGTGTGT	27
Map2k6	CGGCCCACATATCCAGAG<br>ATGGCTTTTTAGTCCCCAAGT	84
Bnip3	GAAGGTTTTCCTTCCATCTCTGT<br>AAGGTGCTAGTGGAAGTTGTCAG	97
Fitm2	AAGCGCAACGTCCTCAAC<br>TTGGTAAGGGCAATGAAAGG	94
Gys2	Taqman assay: Mm00523953_m1	/
Slc2a2	Taqman assay: Mm00446224_m1	/
Insr	Taqman assay: Mm01211875_m1	/
Ppargc1α	Taqman assay: Mm01208835_m1	/
Pklr	Taqman assay: Mm00443090_m1	/
Eef2	Taqman assay: Mm01171434_g1	/
